# Quality of Life and Associated Factors in Primary Caregivers of Children with Refractory Epilepsy on Long-Term Ketogenic Diet: A Cross-Sectional Study

**DOI:** 10.3390/healthcare14121761

**Published:** 2026-06-18

**Authors:** Xia Li, Juan Wang, Xiaoyan Yi, Qin Deng, Yong Zhao, Yongfang Liu

**Affiliations:** 1 Children’s Hospital of Chongqing Medical University, Chongqing 400014, China; 2022410196@stu.cqmu.edu (X.L.); wangjuannn@126.com (J.W.); 15215012925@163.com (X.Y.); 18625934442@163.com (Q.D.); 2 College of Public Health, Chongqing Medical University, Chongqing 401331, China; 3 Ministry of Education Key Laboratory of Child Development and Disorders, Chongqing 400014, China; 4 Department of Nutrition, Children’s Hospital of Chongqing Medical University, Chongqing 400016, China; 5 National Clinical Research Center for Children and Adolescents’ Health and Diseases, Children’s Hospital of Chongqing Medical University, Chongqing 401331, China; 6 Research Center for Medicine and Social Development, Chongqing Medical University, Chongqing 401331, China; 7 Research Center for Public Health Security, Chongqing Medical University, Chongqing 401331, China; 8 Nutrition Innovation Platform-Sichuan and Chongqing, Chongqing 401331, China; 9 Chongqing Key Laboratory of Child Nutrition and Health, Children’s Hospital of Chongqing Medical University, Chongqing 400014, China

**Keywords:** refractory epilepsy, ketogenic diet, primary caregivers, quality of life, influencing factors

## Abstract

**Background/Objectives:** In ketogenic therapy for children with refractory epilepsy—a special patient group—the quality of life of primary caregivers is often overlooked. This study aimed to explore the current state of primary caregivers’ quality of life and identify associated risk factors. **Methods:** A cross-sectional study was conducted from 21 January 2024 to 21 January 2025. A total of 117 primary caregivers of children with refractory epilepsy completed the World Health Organization Quality of Life (WHOQOL)-BREF (26 items) and Adherence questionnaire (6 items). Participants were divided into KD therapy groups (n = 51) and non-KD therapy groups (n = 66) according to the treatment. Factors associated with caregivers’ QoL in the ketogenic treatment were analyzed using the multifactor hierarchical regression. **Results:** There was no significant difference in QoL scores between the KD and non-KD caregiver groups (*p* > 0.05). KD adherence emerged as independently associated with caregivers’ QoL, particularly in the environmental domain (Model 1: β = −0.309, *p* = 0.022; Model 2: β = −0.306, *p* = 0.025). A higher KD cost was significantly associated with a lower social domain score in both models (Model 1: β = −0.285, *p* = 0.032; Model 2: β = −0.286, *p* = 0.034). Model 1 for the environmental domain demonstrated modest explanatory power (Adjusted R^2^ = 0.246, *p* = 0.002). **Conclusions:** These findings underscore the need for clinical support systems to assess and address modifiable stressors early in treatment, including family structure, challenges with ketogenic diet therapy adherence, and financial burden. Such comprehensive evaluation is essential for developing effective and personalized interventions.

## 1. Introduction

Epilepsy is a complex neurological disorder that poses a serious challenge to global public health. According to a report by the WHO, there are approximately 70 million people with epilepsy worldwide, 80% of whom live in low-and middle-income countries [1]. This disease not only severely affects patients’ physical and mental health and quality of life, but also places a heavy financial and caregiving burden on their families and society [2]. The latest epidemiological data indicate that there are more than 9 million people with epilepsy in China, with a prevalence rate of 4–7 per 1000. The number of active cases is as high as 6.5 million, with 450,000 new cases each year, 60% of which occur in childhood [3]. Epilepsy is particularly common among children; the incidence and prevalence of epilepsy among adolescents under the age of 20 in China are on the rise [4]. Approximately 30% of these cases eventually progress to drug-resistant epilepsy [5], posing significant challenges for clinical treatment and management.

The ketogenic diet, a specialized dietary regimen characterized by high fat, low carbohydrate, and moderate protein intake, has been proven to be one of the effective non-pharmacological treatments for drug-resistant epilepsy [6]. This therapy requires extreme discipline and long-term commitment. From precisely calculating nutrient ratios and preparing special meals to closely monitoring blood glucose, blood ketones, and potential side effects, its daily implementation is highly complex and time-consuming. This burden primarily falls on the child’s primary caregiver, usually the parents and particularly the mother. In addition to the stress of providing basic care for the illness, they must also take on the extra responsibility of specialized dietary management. Current research indicates that this highly demanding and specialized role in family health management can easily lead to anxiety, depression, social isolation, and obstacles to career advancement among caregivers, severely impacting their overall QoL [7,8]. Although a significant body of research has focused on the efficacy and safety of the KD for children [9,10], there is a lack of studies examining the QoL of primary caregivers undergoing this specific treatment regimen and the factors influencing it. In particular, there is a lack of empirical research that systematically examines how factors related to treatment, such as costs, challenges to adherence and family support, interact with the various dimensions of caregivers’ QoL (physical, psychological, social and environmental) in the context of ketogenic therapy, Understanding these key influencing factors is essential in the long-term management of ketogenic therapy and for evaluating its overall cost-effectiveness.

This study aims to use a cross-sectional survey to assess the current QoL among primary caregivers of children with refractory epilepsy undergoing long-term KD therapy, with a particular focus on exploring the specific pathways through which factors related to ketogenic therapy influence their QoL. The findings are expected to provide an evidence-based foundation for the development of family-centered, more supportive ketogenic therapy management strategies in clinical practice. This approach seeks to optimize treatment outcomes for children while effectively safeguarding and enhancing the physical and mental health of primary caregivers and the overall well-being of the family, thereby promoting the long-term sustainability of the treatment regimen.

## 2. Materials and Methods

### 2.1. Design

In this cross-sectional study, purposive sampling was used and was conducted by 117 primary caregivers of children with Refractory Epilepsy. Based on treatment, participants were stratified into KD groups (n = 51) and non-KD control groups (n = 66) in the Nutrition and Neurology departments from January to December 2024.

### 2.2. Setting

This study was conducted at the Children’s Hospital of Chongqing Medical University in Chongqing, China.

### 2.3. Participants

This study was conducted among primary caregivers of children with Refractory Epilepsy in the Nutrition and Neurology departments. The inclusion and exclusion criteria are as follows.

Inclusion Criteria:

(1) Children diagnosed with refractory epilepsy according to the International League Against Epilepsy (ILAE) 2010 classification;
(2) Aged ≤ 12 years, and
(3) If receiving KD therapy, there will be a minimum treatment duration of ≥3 months.
(4) Primary caregivers of children with refractory epilepsy, aged ≤ 60 years.

Exclusion Criteria:

(1) Primary caregivers with severe physical or mental illnesses.
(2) Primary caregivers are unable to understand or independently complete the questionnaire.

### 2.4. Sample Size

**Figure 1:**
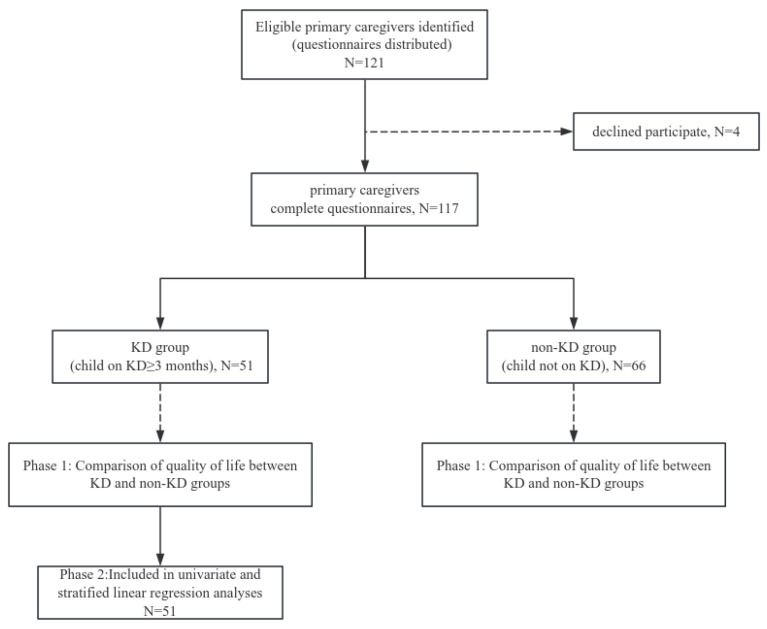
Participant flow diagram.

A total of 121 questionnaires were distributed, of which 117 were returned, and 4 were declined, giving a valid response rate of 96.69%. Of these 51 cases, 51 underwent KD treatment for at least 3 months, constituting the KD group, the focus of this analysis (Figure 1).

### 2.5. Measurements

#### 2.5.1. General Demographic Questionnaire

A self-administered questionnaire collected essential demographic data from the child and the primary caregiver (e.g., gender, age, education level, employment status, health status, family relationship, household income, etc.). KD-cost was assessed using the same questionnaire, defined as the monthly out-of-pocket expenses for ketogenic products only (monitoring supplies, clinic visits, and transportation excluded), estimated by primary caregivers based on recall of the past 30 days.

#### 2.5.2. World Health Organization Quality of Life-BREF (WHOQOL-BREF)

This study applied the WHOQOL-BREF [11,12,13]. The WHOQOL-BREF comprises 26 items (24 domains + 2 global items) grouped into four domains: Physical Health domain (7 items), including assessing physical condition and work capacity; Psychological domain (6 items), including evaluating positive feelings, self-esteem, and negative emotions; Social domain (3 items), including measures of personal relationships and social support satisfaction. Environmental domain (8 items), including safety, housing, financial resources, healthcare access, leisure opportunities, physical environment, and transportation [14]. The scale also includes two core self-rated items: overall health perception and overall QoL. Each item is scored on a 1-to-5 Likert scale, with higher scores indicating better outcomes [15]. Raw scores for each domain are converted to a 4–20 scale or a percentage scale using standardized formulas. Higher total scores indicate a better quality of life.

#### 2.5.3. Parental Adherence Questionnaire

The adherence questionnaire was originally developed by a research team [16]. The form consists of 6 sections: administer an appropriate of the KD as prescribed; meet the daily caloric requirements needed for the KD; provide the diet at the prescribed times; monitor blood glucose and blood ketone levels on time; complete follow-up visits at the ketogenic clinic as scheduled; do not discontinue the KD treatment without authorization and each section is scored as 3 points for complete adherence, 2 points for basic adherence, 1 point for basic failure and 0 points for complete failure. Based on the total score, adherence is classified into three categories: 12 to 18 is considered complete compliance, 6 to 11 is incomplete compliance, and 0 to 5 is complete noncompliance [17]. In the current sample (n = 51), the questionnaire demonstrated acceptable internal consistency (Cronbach’s α = 0.859). The questionnaire was proxy-reported by the primary caregiver.

The full questionnaire is provided in Supplementary Materials S1.

### 2.6. Data Collection

Data were collected by six trained research assistants through face-to-face administration of structured questionnaires in a private environment to ensure confidentiality. All participants provided informed consent. To ensure data quality, the research assistants provided standardized, neutral instructions and were available to clarify items without leading the participants.

### 2.7. Statistical Analysis

Statistical analyses were conducted using SPSS software (version 25.0). Continuous variables were expressed as mean ± standard deviation, while categorical variables were presented as frequencies and percentages. The independent samples *t*-test and ANOVA were used for intergroup comparisons for normally distributed data. The chi-square test was used for frequency comparisons between the two groups. To account for baseline imbalance between groups, analysis of covariance (ANCOVA) was performed. VIF > 10 was excluded from the final model to avoid multicollinearity. Hierarchical linear regression analysis was initially performed to identify potential factors associated with primary caregivers’ QoL. Variables were selected: statistical significance at *p* < 0.10 in the univariate analysis and marginal significance in relation to outcomes of ketogenic therapy. A post-hoc power analysis was performed using G*Power software (version 3.1.9.2) for hierarchical linear regression with 5 predictors and a sample size of 51. With α = 0.05 and assuming a medium effect size (f^2^ = 0.15; Cohen, 1988 [18]), the achieved power (1 − β) was 0.486. For a large effect size (f^2^ = 0.35), the power was 0.886. The values assigned to the respective variables are shown in Table 1. A two-tailed *p*-value < 0.05 was considered statistically significant for all analyses.

## 3. Results

### 3.1. Baseline Characteristics Between Children with Refractory Epilepsy and Their Primary Caregivers in the KD and Control Groups

A total of 121 questionnaires were distributed, with 117 returned, yielding an effective response rate of 96.69%. According to the children’s treatment regimen, the participants comprised 51 primary caregivers from the KD group and 66 from the non-KD group. Baseline characteristics are detailed in Table 2. Among the children, the two groups were comparable in terms of gender, age, and siblings (all *p* > 0.05). The proportion of children residing in urban areas was significantly higher in the KD group (82.4%) than in the non-KD group (63.6%) (*p* = 0.026). The distribution of primary caregivers differed significantly (χ^2^ = 13.007, *p* = 0.001). In the KD group, the primary caregiver was the mother (74.5%), whereas in the non-KD group, it was the father (33.3%). Caregivers in the KD group were more likely to be employed (66.7% vs. 48.5%, *p* = 0.049) and to report a household monthly income of ≥5000 Yuan (58.8% vs. 36.4%, *p* = 0.016). No significant differences were found between groups regarding caregiver age, gender, or educational level (all *p* > 0.05).

### 3.2. Comparison of QoL Among Primary Caregivers Between the KD Group and the Non-KD Group

The baseline characteristics, including primary caregiver, employment, monthly household income, child’s age and residence, were imbalanced between the two groups (all *p* < 0.05). Initial unadjusted comparisons showed no significant differences in QoL across all four domains and the total score (all *p* > 0.05). After adjusting for the identified covariates using ANCOVA, the between-group differences remained non-significant, with *p*-values > 0.05 and partial η^2^ values below 0.011 across all domains. Detailed results are presented in Table 3.

### 3.3. Univariate Analysis of Factors Associated with QOL Among Primary Caregivers in the KD Group

The univariate analysis of factors influencing the QOL among primary caregivers in the KD group demonstrated statistically significant differences (*p* < 0.05) across the physical, psychological, social and environmental domains, with significant variations in the physical domain based on the KD adherence; in the psychological domain and social domain associated with the primary caregivers’ education levels and KD adherence; and in the environmental domain in relation to the children’s siblings, primary caregivers’ educational levels and KD adherence. For detailed results, refer to Table 4.

### 3.4. Hierarchical Linear Regression Analysis Used to Determine the Predictors of the QoL Among Primary Caregivers in the KD Group

The results of the hierarchical multiple regression analysis are summarized in Table 5. Collinearity diagnostics confirmed the absence of multicollinearity, with all VIF values below 10. Predictor variables were selected based on univariate analysis (association with any QoL domain at *p* < 0.10) and clinical rationale.

For the social Domain, in Block 1, a higher KD cost was a significant predictor of a lower social domain score (β = −0.285, *p* = 0.032). The addition of perceived KD efficacy in Block 2 did not lead to a significant increase in explained variance (ΔR^2^ = 0.000, *p* = 0.881). In the final model (Block 2), KD cost remained a significant negative predictor (β = −0.286, *p* = 0.034), while perceived KD efficacy was not significant (β = −0.021, *p* = 0.881). The final model accounted for 25.1% of the variance (R^2^ = 0.251, Adjusted R^2^ = 0.168).

In the environmental domain, in Block 1, a greater number of children’s siblings (β = −0.275, *p* = 0.044) and better KD adherence (β = 0.309, *p* = 0.022) were significant predictors. The inclusion of perceived KD efficacy in Block 2 again resulted in non-significant effects (ΔR^2^ = 0.004, *p* = 0.625). In the final model, the effects of siblings (β = −0.282, *p* = 0.041) and KD adherence (β = 0.306, *p* = 0.025) persisted, and KD efficacy was non-significant (β = −0.067, *p* = 0.625). This model explained 31.0% of the variance (R^2^ = 0.310, Adjusted R^2^ = 0.234).

For the physical and psychological domains, neither the covariates in Block 1 nor the addition of perceived KD efficacy in Block 2 yielded statistically significant results (all *p* > 0.05). For comprehensive results, refer to Table 5.

## 4. Discussion

### 4.1. No Global Differences Between the KD and Non-KD Groups

Previous studies have consistently shown that the QoL of primary caregivers of children with epilepsy is generally impaired [19,20,21]. Research into the QoL of primary caregivers in families receiving ketogenic therapy, a treatment involving significant management, remains insufficient. This study found no statistically significant differences in total QoL scores or scores across various domains between the KD therapy group and the non-KD therapy group. This ‘no difference’ finding may stem from three underlying trade-offs:

First, there is a trade-off between the therapeutic benefits of treatment and the costs of management. While KD is more difficult to manage. It requires strict calculation of dietary ratios and management of adverse reactions. Its efficacy in achieving excellent seizure control in refractory epilepsy (>90% reduction in seizures [22,23]) may constitute a key positive benefit. Once seizures have been significantly reduced and the child’s condition has stabilized, the emotional relief experienced and the reduced caregiving burden may effectively offset the daily burden associated with strict dietary management.

Secondly, consider the prominence of the disease-dominant effect. As all the subjects of this study were families of children with intractable epilepsy, the long-term physical and mental exhaustion, social isolation, and uncertainty about the future caused by the disease itself may constitute the primary ‘background burden’ on the QoL of caregivers [24]. Against this heavy backdrop, any differences resulting from different treatment approaches (ketogenic or non-ketogenic) may be overshadowed by the immense burden of the underlying disease.

Finally, there is the adaptation and reorganization of the family system. Families managing chronic conditions tend to develop new balancing mechanisms under sustained stress [25]. In the KD group, families may achieve a new, treatment-adaptive ’steady state’ through active coping, restructuring of family routines and adjustment of social interaction patterns. This adaptive process may enable them to maintain a level of overall QoL comparable to that of the non-KD therapy group.

Although the study adjusted for baseline imbalances, the association between KD and the primary caregiver’s QoL is not a simple linear relationship. In clinical practice, as children on KD grow older, their awareness of food choices may increase. This heightened autonomy is associated with reduced dietary adherence and may be linked to recurrent seizures; this phenomenon may be associated with a decline in caregivers’ QoL. The non-significant difference in QoL between the two groups may be partially correlated with age-related adherence challenges in the KD group.

### 4.2. The Importance of Adherence, Family Structure and KD Costs Within the KD Group

Although overall scores revealed no significant differences, in-depth analysis identified specific risk factors within the KD group that were significantly associated with lower scores on dimensions of caregiver QoL.

The univariate analysis indicates that educational level, the number of siblings, and adherence to the KD are associated with QoL. A higher educational level may help caregivers better cope with challenges by enhancing their ability to access information, understand scientific concepts, and mobilize resources, and is associated with a higher QoL [26]. In the stratified linear regression analysis, no association was found between educational level and QoL. This is consistent with a partial mediation model, wherein education level may affect QoL partly through its influence on adherence and partly through other unmeasured pathways. Future studies would be needed to formally quantify the indirect effect of education via adherence using mediation analysis.

After adjusting for confounding factors, the stratified linear regression model revealed three independent associated factors.

The Impact of Treatment Adherence on the Environmental Domain: Numerous studies have consistently shown that the success of KD largely depends on strict adherence to it. The unrecognized “hidden costs” of adherence. Adherence may extend beyond simply following a prescribed diet; it may be associated with a state of continuous environmental demands. First, this includes increased financial pressure from purchasing specialized foods and monitoring equipment. Primary caregivers often encounter problems such as the high cost of ketogenic products, product shortages, and the requirement to weigh food [27,28]. Second, the sacrifice of personal time and freedom required for precise meal preparation and detailed journaling. The KD requires the primary caregiver to strictly control the ratio of the three main macronutrients and weigh food ingredients accurately. It also involves keeping a detailed seizure diary and frequently monitoring blood glucose and ketone levels [28]. These treatment procedures can consume a significant amount of the primary caregiver’s personal time. Third, the breakdown of social connections and loss of leisure opportunities resulting from restrictions on dining out and social activities. Young children with epilepsy have limited self-management abilities, so the responsibility for their care falls almost entirely on their parents (particularly mothers). The present study showed that to reduce feelings of shame and avoid accidental risks, caregivers often restrict the children’s activities and social gatherings, as well as limit their own outings and social activities. Studies show that restricting family activities further increases the family’s burden [29]. The chronic psychological strain of managing a child’s food refusal, adverse effects, and dietary conflicts within the family. Adherence to the KD is not achieved overnight; rather, it is an ongoing process that requires overcoming the challenges mentioned above and even more ‘hidden’ ones. An alternative interpretation may also need to be considered. Adherence may itself be a proxy for clinical severity. Because clinical severity, KD duration, AED count and seizure frequency were not measured in this study, we cannot rule out the possibility that the observed associations between adherence and family functioning reflect underlying differences in disease severity rather than the independent effects of adherence per se. Future studies should include direct measures of clinical severity to disentangle these effects.

The Impact of Family Structure on Resources: In the context of ketogenic therapy, which is already a resource-intensive form of care, having multiple children further divides parents’ limited time, energy, and financial resources. Research by D.B. Downey also suggests that resources become diluted as the number of children increases [30]. As primary caregivers have limited time, energy, and financial resources, care may become unevenly distributed. This dilution may affect the ability to create and maintain a stable, structured, and supportive therapeutic environment for the child, which could be associated with a worsening of the existing pressures. Other studies have also shown that having multiple children increases the burden on caregivers [31].

The Association with KD-related Treatment Costs and Social Domain: Higher KD-cost may contribute to the objective financial burden. High out-of-pocket costs not only deplete household financial reserves, but also may be associated with anxiety and feelings of shame, which could lead families to cut back on social spending and might be related to a reduced likelihood of seeking social support. The therapy may be associated with challenges in social relationships and support networks and could contribute to difficulties in the social domain of QoL. A systematic review found that mothers bear the primary burden of caregiving, leading to a significant reduction in working hours and income, resulting in indirect costs accounting for 85% of the total annual burden, and that caregivers commonly experience anxiety, depression, and sleep disorders. S. Yousuf Zafar et al. explained the “economic toxicity” resulting from high out-of-pocket treatment costs, which may reduce QoL [32]. This implies that maintaining and strengthening social support is not only a strategy for coping with the financial demands of KD treatment but also a means to mitigate the erosion of social QoL associated with higher KD treatment-related costs.

This study has some limitations. First, this study was cross-sectional and could not infer a precise causal relationship between the relevant factors and QoL of the primary caregiver, and this relationship still needs to be verified in future prospective studies. Second, the small sample size of this study may affect the stability of the final hierarchical linear regression results. As shown in the post-hoc power analysis, the study was underpowered for medium effect sizes. Non-significant findings for medium effects should be interpreted with caution. Any observed association between adherence and QoL could be partially or fully explained by these unmeasured clinical characteristics. Residual confounding cannot be ruled out. Third, the sample was from a single-center study, and extrapolation of conclusions should be cautious. Future studies should use a longitudinal study design, larger multicenter samples, and incorporate objective indicators for validation.

## 5. Conclusions

This study systematically investigates the multidimensional and complex effects of the KD on the QoL of primary caregivers of children with refractory epilepsy. It also identifies key family risk pathways that extend beyond clinical efficacy.

Research indicates that maintaining a high standard of the KD is not a simple medical procedure, but rather a complex process that requires the reallocation of family resources. Focusing solely on “adherence” while ignoring the associated family costs may result in an incomplete assessment of the treatment’s true impact, potentially leading to caregiver burnout and discontinuation of treatment. Future treatment strategies and support systems should treat the QoL of caregivers and family functioning as core outcome measures of equal importance to seizure frequency. There are two concrete clinical recommendations:

(1) In clinical practice, it is recommended to assess caregiver QoL using a brief, validated instrument (e.g., WHOQOL-BREF) during routine nutrition consultations before KD initiation, as well as at 1, 3 and 6 months after treatment initiation.
(2) Clinicians and dietitians should regularly develop personalized KD meal plans for the child.

This would help to achieve a sustainable balance between ‘seizure control’ and ‘maintaining the family’s overall well-being’.

## Figures and Tables

**Table 1:** Research variable definitions and assignments.

Variable	Explanation of Variable Assignment
Primary caregivers	1 = mother, 2 = father, 3 = other
Age, year	1 = “<30”, 2 = “≥30”
Caregivers’ gender	1 = man, 2 = female
Number of brothers/sisters	0 = “none”, 1 = “≥one”
Children’s gender	1 = man, 2 = female
Educational level	1 = High school or below, 2 = College/university or above
Employment	1 = employed, 2 = Don’t work for epilepsy
Household income, Yuan/month	1 = “<5000”, 2 = “≥5000”
Type of residence	1 = Urban, 2 = Rural
KD-proportion	1 = “KD product proportion ≥ 50%”, 2 =“KD product proportion <50%”
KD-making-difficulty	1 = yes, 2 = no
KD-family-support	1 = all against, 2 = some in favor, 3 = all in favor
KD-effective	1 = effective, 2 = ineffective
KD-side-effects	0 = no, 1 = yes
KD-adherence	1 = not at all, 2 = not fully, 3 = fully adherent
KD-cost, Yuan/month	1 = “<2000”, 2 = “≥2000”

**Table 2:** Comparison of Baseline Characteristics Between Children with Epilepsy and Their Primary Caregivers in the KD and Non-KD Groups.

Variable	KD Group	Non-KD Group	*t*/χ^2^	*p*
Primary caregivers, n (%)			13.007	**0.001**
Mother	38 (74.5)	36 (54.5)		
Father	3 (5.9)	22 (33.3)		
Other	10 (19.6)	8 (12.1)		
Age, year, n (%)			1.042	0.307
<30	14 (27.5)	24 (36.4)		
≥30	37 (72.5)	42 (63.6)		
Caregivers’ gender, n (%)			2.348	0.125
Male	6 (11.8)	15 (22.7)		
Female	45 (88.2)	51 (77.3)		
Educational level, n (%)			0.167	0.683
High school or below	29 (56.9)	40 (60.6)		
College/university or above	22 (43.1)	26 (39.4)		
Employment, n (%)			3.868	**0.049**
employed	34 (66.7)	32 (48.5)		
Don’t work for epilepsy	17 (33.3)	34 (51.5)		
Household income, Yuan/month, n (%)			5.840	**0.016**
<5000	21 (41.2)	42 (63.6)		
≥5000	30 (58.8)	24 (36.4)		
Children’s gender, n (%)			0.214	0.644
Male	30 (58.8)	36 (54.5)		
Female	21 (41.2)	30 (45.5)		
Age, month (mean ± SD)	73.47 ± 37.418	49.24 ± 43.370	3.170	**0.002**
Having brothers/sisters, n (%)			0.018	0.892
no	21 (41.2)	28 (42.4)		
yes	30 (58.8)	38 (57.6)		
Type of residence			4.977	**0.026**
Urban	42 (82.4)	42 (63.6)		
Rural	9 (17.6)	24 (36.4)		

Bold *p*-values indicate statistical significance at *p* < 0.05.

**Table 3:** Comparison of WHOQOL-BREF Domain Scores Between Primary Caregivers in the KD Group and the Non-KD Group ($\bar{x}$ ± SD).

Group	Physiological Domains	Psychological Domains	Social Domains	Environmental Domains	T-Score
KD Group (n = 51, $\bar{x}$ ± s)	12.58 ± 2.09	12.21 ± 2.60	13.7 ± 3.15	12.39 ± 3.02	12.72 ± 2.51
Epilepsy Group (n = 66, $\bar{x}$ ± s)	12.98 ± 2.25	12.73 ± 2.60	13.96 ± 3.16	12.61 ± 3.08	13.07 ± 2.54
Unadjusted Difference (95% CI)	−0.396 (−1.200, 0.409)	−0.518 (−1.480, 0.444)	−0.260 (−1.424, 0.904)	−0.214 (−1.342, 0.914)	−0.347 (−1.279, 0.585)
*t*(*p*)	−0.974 (0.332)	−1.067 (0.288)	−0.443 (0.659)	−0.376 (0.708)	−0.736 (0.463)
KD Group (n = 51, adjusted $\bar{x}$ ± s)	12.66 ± 0.32	12.17 ± 0.39	13.73 ± 0.47	12.37 ± 0.46	12.73 ± 0.38
Epilepsy Group (n = 66, adjusted $\bar{x}$ ± s)	12.92 ± 0.28	12.76 ± 0.34	13.94 ± 0.41	12.62 ± 0.40	13.06 ± 0.33
Adjusted Difference (95% CI)	−0.255 (−1.147, 0.636)	−0.589 (−1.666, 0.489)	−0.213 (−1.511, 1.086)	−0.250 (−1.515, 1.014)	−0.327 (−1.371, 0.718)
*F*(*p*)	0.322 (0.572)	1.171 (0.281)	0.105 (0.746)	0.154 (0.696)	0.384 (0.537)
Bias η^2^	0.003	0.011	0.001	0.001	0.003

**Table 4:** Univariate Analysis of Factors Associated with Quality of Life in Primary Caregivers of the KD Group.

Variables	Physiological Domains		Psychological Domains		Social Domains		Environmental Domains	
	*t*/*F*	*p*	*t*/*F*	*p*	*t*/*F*	*p*	*t*/*F*	*p*
Primary caregivers	0.20	0.819	1.25	0.296	1.70	0.193	0.44	0.647
Caregivers’ age	1.54	0.130	0.16	0.875	−0.11	0.912	−1.11	0.273
Caregivers’ gender	0.22	0.826	−0.21	0.837	1.56	0.125	0.52	0.604
Number of brothers/sisters	1.30	0.200	1.28	0.208	1.75	0.086	2.73	**0.009**
Children’s age	0.55	0.463	2.65	0.110	0.72	0.400	1.47	0.231
Children’s gender	−0.28	0.783	−0.60	0.551	−1.12	0.270	−0.68	0.499
Educational level	−1.29	0.203	−2.18	**0.034**	−2.24	**0.030**	−2.23	**0.031**
Employment	1.09	0.283	0.70	0.488	0.84	0.407	0.80	0.427
Household income	−0.41	0.684	0.25	0.807	−0.09	0.928	−0.44	0.660
Urban/rural residence	−1.30	0.201	−0.96	0.344	−0.24	0.814	0.43	0.672
KD-proportion	−0.75	0.459	−1.10	0.276	−0.49	0.629	−0.96	0.343
KD-making-difficulty	−0.84	0.408	−0.98	0.334	−0.63	0.528	−0.92	0.362
KD-family-support	2.00	0.146	0.77	0.470	0.61	0.550	0.63	0.540
KD-effective	0.97	0.339	1.52	0.136	0.92	0.364	1.12	0.268
KD-side-effects	−0.34	0.733	−0.59	0.560	−0.03	0.979	0.01	0.996
KD-adherence	2.31	**0.025**	2.33	**0.024**	2.47	**0.017**	2.98	**0.004**
KD-cost	0.61	0.546	1.08	0.286	1.78	0.081	1.37	0.178

Bold *p*-values indicate statistical significance at *p* < 0.05.

**Table 5:** Hierarchical linear regression analysis of QoL among primary caregivers in the ketogenic diet.

	Physiological Domains		Psychological Domains		Social Domains		Environmental Domains		Collinearity Statistics
	β (95% CI)	*p*	β (95% CI)	*p*	β (95% CI)	*p*	β (95% CI)	*p*	VIF
Block 1									
Having brothers/sisters	−0.105 (−1.697, 0.817)	0.484	−0.072 (−1.897, 1.142)	0.619	−0.153 (−2.720, 0.790)	0.274	−0.275 (−3.288, −0.050)	0.044	1.165
Educational level	0.067 (−0.972, 1.535)	0.653	0.200 (−0.476, 2.554)	0.174	0.175 (−0.649, 2.851)	0.212	0.125 (−0.864, 2.366)	0.354	1.174
KD-adherence	−0.279 (−2.517, 0.066)	0.062	−0.257 (−2.966, 0.155)	0.076	−0.270 (−3.583, 0.022)	0.053	−0.309 (−3.623, −0.296)	0.022	1.128
KD-cost	−0.122 (−1.741, 0.685)	0.385	−0.179 (−2.433, 0.499)	0.191	−0.285 (−3.551, −0.163)	0.032	−0.248 (−3.115, 0.011)	0.052	1.023
Block 1 R^2^	0.129		0.181		0.250		0.307		
Block 1 Adjusted R^2^	0.054		0.109		0.185		0.246		
F(*p*)	1.711 (0.164)		2.536 (0.053)		3.841 (0.009)		5.083 (0.002)		
Block 2									
Having brothers/sisters	−0.113 (−1.752, 0.798)	0.455	−0.084 (−1.977, 1.095)	0.566	−0.155 (−2.765, 0.806)	0.275	−0.282 (−3.356, −0.069)	0.041	1.179
Educational level	0.033 (−1.240, 1.514)	0.842	0.151 (−0.873, 2.446)	0.345	0.166 (−0.885, 2.972)	0.282	0.096 (−1.196, 2.354)	0.515	1.393
KD-adherence	−0.275 (−2.513, 0.094)	0.068	−0.252 (−2.948, 0.193)	0.084	−0.269 (−3.600, 0.051)	0.056	−0.306 (−3.621, −0.260)	0.025	1.130
KD-cost	−0.124 (−1.761, 0.687)	0.381	−0.182 (−2.458, 0.491)	0.186	−0.286 (−3.575, −0.147)	0.034	−0.250 (−3.141, 0.014)	0.052	1.024
KD-effective	−0.081 (−1.616, 0.940)	0.597	−0.114 (−2.129, 0.950)	0.444	−0.021 (−1.924, 1.656)	0.881	−0.067 (−2.050, 1.245)	0.625	1.211
Block 2 R^2^	0.135		0.191		0.251		0.316		
Block 2 Adjusted R^2^	0.039		0.102		0.168		0.234		
F(*p*)	1.404 (0.241)		2.130 (0.079)		3.012 (0.020)		4.048 (0.004)		

## Data Availability

The data presented in this study are available on reasonable request from the corresponding author. The data are not publicly available due to privacy and ethical restrictions established by the Children’s Hospital of Chongqing Medical University, as they contain information that could compromise the confidentiality of study participants.

## Supplementary Materials

The following supporting information can be downloaded at: https://www.mdpi.com/article/10.3390/healthcare14121761/s1, Supplementary Materials S1: Quality of Life Scale for Children’s Primary Caregivers; Supplementary Materials S2: Review and Approval of the Ethics Committee of Children’s Hospital Affiliated to Chongqing Medical University.

## Abbreviations

The following abbreviations are used in this manuscript:

(WHOQOL)-BREF	World Health Organization Quality of Life-BREF
QoL	Quality of Life
KD	Ketogenic Diet
ILAE	International League Against Epilepsy
