# Supplementary material for: Quality of Life and Associated Factors in Primary Caregivers of Children with Refractory Epilepsy on Long-Term Ketogenic Diet: A Cross-Sectional Study
Source: Healthcare (Basel). 2026 Jun 18;14(12):1761. doi: 10.3390/healthcare14121761 (PMC13299362; doi:10.3390/healthcare14121761)
Supplement: Supplementary file 1 [file healthcare-14-01761-s001.zip › healthcare-4225540-supplementary material 1.pdf]

Quality of Life Scale for Children's Primary Caregivers

Dear Parents and Children:
Greetings! We would like to invite you to participate in the "Survey on Quality of Life of Caregivers of Children 0-12 Years of Age" conducted by Children's Hospital of Chongqing Medical University. The following questions contain basic information about you and your child and your views on quality of life, health or other aspects of life. Please select the most appropriate answer for each question. If you are not sure at the moment, the first response in your mind is often the most correct. For all questions, please answer according to your own standards, wishes or feelings. Note that all questions are about your situation in **the last 4 weeks**.

The primary caregiver (the person who spends the most time with the child and performs the most caregiving tasks) filled out the questionnaire anonymously. There were no right or wrong answers, and the survey was mainly used for scientific research and analysis.

The ketogenic diet, which is high in fat and low in carbohydrates, is an important treatment for refractory epilepsy. It is one of the most important treatment modalities for refractory epilepsy. This study analyses the quality of life and influencing factors of the primary caregivers of children with refractory epilepsy on the ketogenic diet. The aim is to provide a theoretical basis for improving the quality of life of families with children with refractory epilepsy. This survey is completely confidential and will not affect you in any way, so please feel free to complete it. (This survey should take about **5–10 minutes**. Thank you for your cooperation and support.)

Informed consent: I have read the above information, and I understand it. I am participating in this survey voluntarily.
○Yes (skip to first part of question)
○No (end of answer)

Setion 1
basic imformation
1. **The child's primary caregiver is:** ①father ②mother ③other
2. **Primary caregiver's age is:__________** ①<30year ②>=30year
3. **Primary caregiver's gender:** ①man ②woman
4. **Children's siblings:** ①no ②have
5. **The age of the child is: _______years _______months**
6. **Child's gender:** ①man ②woman
7. **The child's current height (length) is_______(cm) and weight is _______(kg).**
8. **Age at disease diagnosis: _______years_______ months**
9. **The number of drugs this child now ingests: _____**
10. **Is the child on a ketogenic diet**: ①Yes ②No ("Yes" skip to next question, "No" skip to question 21.)
11. **At the time of the child's first ketogenic diet, the child's age was: _______years _______months**
12. **the proportion of the child's primary food sources on the ketogenic diet:**

① 100% ketogenic products
② 90% ≤ ketogenic products <100%, or 0% <food meals <10%
③ 50% ≤ ketogenic products <90%, or 10% ≤food meals <50%
④ 10% ≤ ketogenic products <50%, or 50% ≤food meals <90%
⑤ 0% <ketogenic products <10%, or 90% ≤food meals <100%
⑥ 100% ketogenic meal share

13. **Is meal preparation on the ketogenic diet difficult for you and your family:**

① Yes ② No

14. **Opinions of your family members about ketogenic diet for your child:**

① All against

② Some in favor
③ All in favor

15. How long has the child been on the ketogenic diet:

① 0-3month
② 4-6month
③ 7-9month
④ 10-12month
⑤ >12month

16. The frequency of disease episodes in this child after ketogenic diet treatment (clinical efficacy Engel grading, 1-3 effective, 4 ineffective):

① no episodes, 100% reduction in episodes
② 90%-99% reduction in episodes
③ 50%-90% reduction in episodes
④ <50% reduction in episodes

17. Whether the EEG of the child improved after ketogenic diet treatment:

①Yes ②No

18. Whether the child's cognitive function improved after the ketogenic diet:

①Yes ②No

19. did this child have any of the following reactions after the ketogenic diet:

○ None
○ Gastrointestinal disorders (e.g., constipation, diarrhea, etc.)
○ Acute pancreatitis
○ Hypoproteinemia
○ Hyperlipidemia
○ Calcification of renal function
○ Calcium loss in bone
○ Lagging/tardy growth
○ Other

20. Ketogenic diet treatment adherence

	Fully achieved	Basically achieved	Basically unachieved	Completely unachieved
Ketogenic diet in the right proportions as prescribed by the doctor				
Calories needed to complete the daily ketogenic diet				
Diets are given at the prescribed time				
Monitor blood glucose and ketone levels on a regular basis				
Timely completion of ketogenic clinic follow-up visits				
Not stopping ketogenic diet therapy without authorization				

21. **Your education level:** ① High school or below ② College/university or above
22. **Your health condition:** ① poor ② average ③ good ④ very healthy
23. **Your work situation:** ①Yes ②No (Because of caring for sick children)
24. **Your monthly household income:** ○<5000 Yuan ○>=5000
25. **Your child's average total monthly spending on treatment:** ○<5000 Yuan ○>=5000
26. **Average monthly consumption of ketogenic diet therapy only:** ○<2000 Yuan ○>=2000(Completed by the primary caregiver of the child treated with the ketogenic diet)
27. **Your place of residence:** ○Urban ○ Rural

Section 2

**You would circle number 1 if you did not get any of the support that you needed from others in the last two weeks. Please read each question, assess your feelings, and circle the number on the scale for each question that gives the best answer for you.**						
		**Very poor**	**Poor**	**Neither poor nor good**	**Good**	**Very good**
**1.**	**How would you rate your quality of life?**	**1**	**2**	**3**	**4**	**5**
		**Very dissatisfied**	**Dissatisfied**	**Neither satisfied nor dissatisfied**	**Satisfied**	**Very satisfied**
**2.**	**How satisfied are you with your health?**	**1**	**2**	**3**	**4**	**5**
**The following questions are about your feelings about experiencing certain things in the past 4 weeks**						
		**not at all**	**A little**	**A moderate amount**	**Very much**	**An extreme amount**
**3.**	How bothered are you by physical pain that prevents you from doing what needs to be done?	**5**	**4**	**3**	**2**	**1**
**4.**	How much do you need any medical treatment to function in your daily life?	**5**	**4**	**3**	**2**	**1**
**5.**	How much do you enjoy life?	**1**	**2**	**3**	**4**	**5**
**6.**	To what extent do you feel your life to be meaningful?	**1**	**2**	**3**	**4**	**5**
		**Not at all**	**A little**	**A moderate amount**	**Very much**	**Extremely**
**7.**	How well are you able to concentrate?	**1**	**2**	**3**	**4**	**5**
**8.**	How safe do you feel in your daily life?	**1**	**2**	**3**	**4**	**5**
**9.**	How healthy is your physical environment?	**1**	**2**	**3**	**4**	**5**
**The following questions ask about how completely you experience or were able to do certain things in the last two weeks.**						
		**Not at all**	**A little**	**Moderately**	**Mostly**	**Completely**
**10.**	Do you have enough energy for everyday life?	**1**	**2**	**3**	**4**	**5**
**11.**	Are you able to accept your bodily appearance?	**1**	**2**	**3**	**4**	**5**
**12.**	Have you enough money to meet your needs?	**1**	**2**	**3**	**4**	**5**
**13.**	How available to you is the information that you need in your day-to-day life?	**1**	**2**	**3**	**4**	**5**
**14.**	How well are you able to get around?	**1**	**2**	**3**	**4**	**5**
		**Very poor**	**Poor**	**Neither poor nor**	**Good**	**Very good**

				**good**		
**15.**	How well are you able to get around?	**1**	**2**	**3**	**4**	**5**
**The following questions ask you to say how good or satisfied you have felt about various aspects of your life over the last two weeks.**						
		**Very dissatisfied**	**Dissatisfied**	**Neither satisfied nor dissatisfied**	**Satisfied**	**Very satisfied**
**16.**	How satisfied are you with your sleep?	**1**	**2**	**3**	**4**	**5**
**17.**	How satisfied are you with your ability to perform your daily living activities?	**1**	**2**	**3**	**4**	**5**
**18.**	How satisfied are you with your capacity for work?	**1**	**2**	**3**	**4**	**5**
**19.**	How satisfied are you with yourself?	**1**	**2**	**3**	**4**	**5**
**20.**	How satisfied are you with your personal relationships?	**1**	**2**	**3**	**4**	**5**
**21.**	How satisfied are you with your sex life?	**1**	**2**	**3**	**4**	**5**
**22.**	How satisfied are you with the support you get from your friends?	**1**	**2**	**3**	**4**	**5**
**23.**	How satisfied are you with the conditions of your living place?	**1**	**2**	**3**	**4**	**5**
**24.**	How satisfied are you with your access to health services?	**1**	**2**	**3**	**4**	**5**
**25.**	How satisfied are you with your transport?	**1**	**2**	**3**	**4**	**5**
**The following question refers to how often you have felt or experienced certain things in the last two weeks.**						
		**Never**	**Seldom**	**Quite often**	**Very often**	**Always**
**26.**	How often do you have negative feelings such as blue mood, despair, anxiety, depression?	**5**	**4**	**3**	**2**	**1**
**Did someone help you to fill out this form?**						
**How long did it take to fill this form out?**						

World Health Organization Quality of Life(WHOQOL)-BREF

This concludes this questionnaire! For the assistance you have provided, we sincerely thank you!
